# The impact of cerebellar transcranial direct current stimulation (tDCS) on sensorimotor and inter-sensory temporal recalibration

**DOI:** 10.3389/fnhum.2022.998843

**Published:** 2022-08-30

**Authors:** Christina V. Schmitter, Benjamin Straube

**Affiliations:** ^1^Department of Psychiatry and Psychotherapy, University of Marburg, Marburg, Germany; ^2^Center for Mind, Brain and Behavior, University of Marburg and Justus Liebig University Giessen, Marburg, Germany

**Keywords:** sensorimotor temporal recalibration, sensorimotor adaptation, predictive processing, forward model, cerebellum, transcranial direct current stimulation, tDCS

## Abstract

The characteristic temporal relationship between actions and their sensory outcomes allows us to distinguish self- from externally generated sensory events. However, the complex sensory environment can cause transient delays between action and outcome calling for flexible recalibration of predicted sensorimotor timing. Since the neural underpinnings of this process are largely unknown this study investigated the involvement of the cerebellum by means of cerebellar transcranial direct current stimulation (ctDCS). While receiving anodal, cathodal, dual-hemisphere or sham ctDCS, in an adaptation phase, participants were exposed to constant delays of 150 ms between actively or passively generated button presses and visual sensory outcomes. Recalibration in the same (visual outcome) and in another sensory modality (auditory outcome) was assessed in a subsequent test phase during which variable delays between button press and visual or auditory outcome had to be detected. Results indicated that temporal recalibration occurred in audition after anodal ctDCS while it was absent in vision. As the adaptation modality was visual, effects in audition suggest that recalibration occurred on a supra-modal level. In active conditions, anodal ctDCS improved sensorimotor recalibration at the delay level closest to the adaptation delay, suggesting a precise cerebellar-dependent temporal recalibration mechanism. In passive conditions, the facilitation of inter-sensory recalibration by anodal ctDCS was overall stronger and tuned to larger delays. These findings point to a role of the cerebellum in supra-modal temporal recalibration across sensorimotor and perceptual domains, but the differential manifestation of the effect across delay levels in active and passive conditions points to differences in the underlying mechanisms depending on the availability of action-based predictions. Furthermore, these results suggest that anodal ctDCS can be a promising tool for facilitating effects of temporal recalibration in sensorimotor and inter-sensory contexts.

## Introduction

Despite the multitude of sensory signals that we are exposed to during daily interactions with the environment, we can effortlessly distinguish between those caused by our own actions and those with external origin. This ability relies heavily on the characteristic and highly predictable temporal relationship between actions and their sensory outcomes. For example, when clapping hands, the clap sound is strongly expected to be perceived near-instantaneously after the hands touch, by considering inherent delays in sound transmission and in sensory pathways. Such predictions about sensory action-outcomes are assumed to be generated by an internal forward model based on a copy of the motor commands (Blakemore et al., 1998; Wolpert et al., 1998; Elijah et al., 2016; Cao et al., 2017). Sensations that occur with the timing predicted for the action-outcome are likely to be attributed to the own action. A temporal mismatch, however, such as an unexpected long delay between action and outcome, elicits a prediction error and the attribution of sensations to an external event or agent (Haggard et al., 2002; Hughes et al., 2013; Imaizumi and Tanno, 2019; Zapparoli et al., 2020).

Notably, the complexity of the sensory environment can cause transient violations in the temporal relationship between action and outcome. For instance, additional delays can be imposed on the interval between an action and the perception of its outcome by changes in environmental conditions (e.g., dimmed light delaying signals from the retina; Matteson, 1971) or by changes in sensory processing (e.g., due to fatigue; Cai et al., 2018). Thus, it seems essential to dynamically recalibrate predictions about the timing of sensory action-outcomes to preserve accurate perception and attribution of agency even under frequently changing conditions (Stetson et al., 2006; Parsons et al., 2013; Cai et al., 2018). Such a recalibration of perception also has direct implications for motor functions, which have to be adapted to the changed temporal dynamics as well. An important everyday example is locomotion. Here, the adequate adaptation of temporal gait characteristics, e.g., step timing, to varying environmental conditions is an important indicator of the successful control of limb positions during locomotion (Hoogkamer and O’Brien, 2016; Gonzalez-Rubio et al., 2019).

Indeed, flexible recalibration of the perceived relative timing between actions and their sensory outcomes is an established phenomenon known as sensorimotor temporal recalibration. Evidence for this phenomenon can be derived from a range of studies that aimed at inducing a sensorimotor temporal recalibration effect (TRE) by repeatedly inserting a constant delay between a participant’s action (like a button press) and its sensory outcome in form of a light flash or a brief tone (Stetson et al., 2006; Heron et al., 2009; Sugano et al., 2010, 2012, 2016, 2017; Stekelenburg et al., 2011; Tsujita and Ichikawa, 2012; Rohde and Ernst, 2013; Elijah et al., 2016; Cao et al., 2017; Cai et al., 2018; Arikan et al., 2021). After repeated exposure to such a manipulation, the delayed action-outcome was in fact perceived as occurring synchronously with the action (Sugano et al., 2010, Keetels and Vroomen, 2012; Sugano et al., 2012, 2016, 2017; Yamamoto and Kawabata, 2014) and shorter delays were less likely to be detected (Arikan et al., 2021). This indicates recalibration of the expected relative timing between action and outcome leading to a shift of synchrony perception toward the exposed delay. Moreover, undelayed outcomes were now frequently perceived as preceding the action (Stetson et al., 2006; Heron et al., 2009; Sugano et al., 2010; Stekelenburg et al., 2011; Tsujita and Ichikawa, 2012; Rohde and Ernst, 2013; Cai et al., 2018) suggesting that the constant delay was incorporated into the temporal prediction of the outcome.

Notably, while sensorimotor temporal recalibration finds support in a wide range of behavioral studies, evidence for the neural basis of this process remains sparse. The sensorimotor TRE could for instance be associated with in a shift of readiness potentials closer to movement onset indicating the involvement of action-based predictive mechanisms (Cai et al., 2018). Moreover, the illusory perception of an undelayed or very shortly delayed outcome as preceding the action after exposure to a constant delay was related to increased hemodynamic activation of brain areas involved in error-related processing such as anterior cingulate cortex and medial frontal cortex (Stetson et al., 2006). However, while these findings describe correlates of the sensorimotor TRE in the brain, the neural mechanisms behind the process itself remain largely elusive.

The cerebellum has emerged as an important brain area regarding the generation of predictions about sensory action-outcomes. Since it receives and combines afferent inputs from sensory areas and efferent inputs from motor areas, its anatomy and location seem ideal for performing predictive forward model computations. It has consequently been proposed as critical brain structure for forward model related processes or even as the site of internal forward models itself (Miall et al., 1993; Wolpert et al., 1998; Imamizu et al., 2000; Blakemore et al., 2001; Ishikawa et al., 2016; Cao et al., 2017; Straube et al., 2017b; Arikan et al., 2019; van Kemenade et al., 2019; Tanaka et al., 2020; Welniarz et al., 2021). It is further well-known to be involved in potentially related processes such as motor control, adaptation and learning (Tseng et al., 2007; Synofzik et al., 2008; Shadmehr et al., 2010; Schlerf et al., 2012; Sokolov et al., 2017; Statton et al., 2018; Tanaka et al., 2020). This suggests that the cerebellum could also be a prime candidate area for the updating of action-outcome predictions and thus for the process of sensorimotor temporal recalibration.

It could be argued that the effects of sensorimotor temporal recalibration emerge simply due to recalibration of the perceived inter-sensory timing (e.g., between the tactile sensation at the end of the button press and the visual or auditory outcome) which is also known to be an important mechanism for dealing with differential and varying delays in the transmission and processing of sensations from different sensory modalities (Fujisaki et al., 2004; Vroomen et al., 2004; Hanson et al., 2008; van der Burg et al., 2013). Importantly however, the TRE appeared to be stronger when the action was self-initiated compared to conditions where the effector was passively touched (Stetson et al., 2006) or moved (Arikan et al., 2021). Since a TRE in such passive conditions can only be explained by inter-sensory temporal recalibration due to the absence of a motor command or the intention to act, the weaker effect in this condition points to a component in sensorimotor temporal recalibration that is specific to the processing of sensorimotor delays and can indeed be attributed to the adaptation of action-based predictions (Arikan et al., 2021).

These results also suggests that the involvement of regions known for action-outcome processing, such as the cerebellum, in temporal recalibration is specific to the sensorimotor context, but direct evidence for this claim is missing.

A recent MEG study reported first evidence for cerebellar contributions to temporal recalibration by investigating the M100 component known to reflect an early response to auditory stimuli. This component is typically attenuated for the processing of tones that occur in synchrony with a self-generated action compared to passive listening to externally presented tones. Here, after repeated exposure to a delayed tone, M100 attenuation also emerged for the delayed tones, but this effect was abolished after inhibition of the right cerebellum by TMS (Cao et al., 2017). While this points to a vital role of the cerebellum in temporal recalibration, it remains unclear whether this effect is indeed specific to sensorimotor as opposed to more general audio-tactile temporal recalibration and whether it can be linked to relevant changes in behavior.

Interestingly, the sensorimotor TRE could frequently be shown to transfer from one modality to another, such that the temporal perception of the action-outcome of one modality recalibrated to a delay previously inserted between the action and the outcome of another modality (Heron et al., 2009; Sugano et al., 2010, 2012; Arikan et al., 2021). This suggests that sensorimotor temporal recalibration does not occur in modality-specific circuits but rather on a supra-modal level, i.e., in the general predicted timing for sensory outcomes following an action. This assumption coincides with evidence that action-outcome predictions are simultaneously generated by the internal forward model for outcomes in multiple sensory modalities (van Kemenade et al., 2016, 2017; Straube et al., 2017b). Whether the neural correlates for the updating of action-outcome predictions, which might be assumed in the cerebellum, also perform this updating on a supra-modal level and are therefore responsible for the modality-transfer effects, remains to be determined.

In this study, we therefore used transcranial direct current stimulation (tDCS) on the cerebellum to address the question of whether it is related to temporal recalibration. We were specifically interested in the question of whether the relationship is unique to the sensorimotor context and whether the cerebellum contributes to the modality-transfer of recalibration effects. tDCS is a non-invasive brain stimulation technique that has already been used by a range of previous studies that investigated whether the stimulation of mainly frontal and parietal brain areas can influence the processing of sensory action outcomes. Frontal tDCS for instance facilitated the detection of delays between an action and its sensory outcome (Straube et al., 2017a,^2020^) while stimulation of the pre-supplementary motor area (Cavazzana et al., 2015), angular gyrus (Khalighinejad and Haggard, 2015) or dorsolateral prefrontal cortex (Khalighinejad et al., 2016) modulated the intentional binding effect, i.e., an implicit measure for the perceived agency over a sensory event. Furthermore, visual cortex tDCS was able to influence the extent of the visuomotor TRE (Aytemür et al., 2017). The efficacy of cerebellar tDCS (ctDCS) has been shown for a variety of processes as well, e.g., for the modulation of motor learning (Shah et al., 2013; Celnik, 2015; Shimizu et al., 2017; Kumari et al., 2019) and adaptation (Jayaram et al., 2012; Doppelmayr et al., 2016; Yavari et al., 2016; Panico et al., 2018; Weightman et al., 2021), balance control (Ehsani et al., 2017) or procedural learning (Ferrucci et al., 2013; Gupta et al., 2018). Despite the presumable critical importance of the cerebellum for the predictive mechanisms underlying action-outcome processing, evidence for the impact of ctDCS in this context and especially when recalibration of predictions is necessary due to repeated temporal action-outcome deviations is missing. Here, we tested if and to what extent ctDCS of different polarities can facilitate sensorimotor temporal recalibration.

Participants engaged in adaptation phases during which the temporal relationship between a button press and a visual sensory outcome was manipulated by introducing constant delays between button press and outcome. A subsequent delay detection task assessed if and to what extent these temporal incongruencies triggered temporal recalibration. Here, participants were asked to detect varying temporal delays between the button press and its sensory outcomes. The TRE was expected to manifest in decreased delay detection performance (especially for delays close to the adaptation delay) indicating a shift of predicted stimulus timing in the direction of the constant delay participants were previously exposed to. Importantly, button presses were either performed actively by the participants or passively by an electromagnetic passive button device. Since both passive and active movements were associated with similar tactile and proprioceptive sensations, this manipulation allowed us to disentangle effects of sensorimotor temporal recalibration due to adaptation of action-based predictions from effects due to inter-sensory temporal recalibration. Based on previous findings we expected a stronger TRE in active compared to passive movement conditions due to the additional involvement of predictive signals based on the motor commands (Stetson et al., 2006; Arikan et al., 2021). As actively compared to passively elicited sensory stimuli could previously be associated with generally enhanced delay detection performance (van Kemenade et al., 2016), an increased TRE due to the availability of action-based predictions could for example reflect an advantage in processing temporal prediction errors.

While the constant delay was always inserted between button presses and visual outcomes during adaptation (visuomotor or visuo-tactile temporal recalibration), the sensory modality in the delay detection task could be either visual or auditory. Based on the assumption that action-outcome predictions are generated for multiple sensory modalities (van Kemenade et al., 2016, 2017; Straube et al., 2017b) and based on previous observations (Heron et al., 2009; Sugano et al., 2010, 2012; Arikan et al., 2021) we expected the visuomotor or visuo-tactile temporal recalibration procedure to induce a TRE for both visual (visuomotor or visuo-tactile TRE) and auditory test modalities (audiomotor or audio-tactile TRE) and particularly so in active movement conditions due to recalibration of predictions on a supra-modal level.

This experimental paradigm was applied in four separate sessions for each participant during which they received 20 min of either anodal, cathodal, dual-hemisphere or sham ctDCS to investigate whether the TRE can be facilitated or impaired, respectively, depending on stimulation polarity, which might be attributed to changes in the sensitivity to temporal prediction errors or in the speed or precision of sensorimotor learning.

Anodal tDCS has frequently been shown to increase cortical excitability while it is decreased by cathodal tDCS (Nitsche and Paulus, 2000). Although such polarity-dependent effects seem to be more inconsistent for the cerebellum, similar directions of the effect have often been reported here as well (Grimaldi et al., 2016). Therefore, compared to sham stimulation, we expected temporal recalibration to be facilitated by anodal ctDCS on the bilateral cerebellum but to be impaired by cathodal ctDCS. Since dual-hemisphere tDCS could be demonstrated to increase stimulation effects (Vines et al., 2008; Kwon and Jang, 2012; Workman et al., 2020), presumably due to reduced inhibitory inter-hemispheric influences (Kwon and Jang, 2012), we furthermore explored whether stronger faciliatory effects on the TRE could be achieved by simultaneous anodal ctDCS of the right and cathodal ctDCS of the left cerebellar hemisphere compared to purely anodal ctDCS. Since we assumed ctDCS to influence action-based predictive mechanisms located in the cerebellum we expected greater polarity-dependent modulations of the TRE and its modality-transfer to occur in active movement conditions.

## Materials and methods

### Participants

Twenty-two right-handed healthy volunteers participated in the study (10 male; mean age: 25.18 years, *SD* = 4.59). All participants had normal or corrected-to-normal vision and normal hearing. They reported no history of psychiatric, neurological or movement disorders or of drug or alcohol abuse. Additionally, no one reported any contraindications for tDCS (e.g., electric, or metallic implants). According to a power analysis, this sample size should have been sufficient to reproduce effects of similar size as reported in previous studies with a similar experimental design (see section 1 in Supplementary material for further details). Participants provided written informed consent and received financial reimbursement for their participation. The study was conducted according to the Declaration of Helsinki and was approved by the local ethics commission of the medical faculty of University of Marburg, Germany.

### Transcranial direct current stimulation

In each session, ctDCS was applied on the cerebellum with a DC stimulator (neuroConn GmbH, Ilmenau) and two rubber electrodes (5 × 7 cm) covered in saline-soaked sponges (0.9% NaCl).

For anodal and cathodal ctDCS, the center of the respective active electrode was placed on the midline 2 cm below the inion to target the bilateral cerebellum while the return electrode was attached to the right upper arm (onto the deltoid muscle). For dual-hemisphere ctDCS, electrodes were placed with their centers 2 cm below and 3 cm lateral to the inion targeting the right (anode) and the left cerebellar hemisphere (cathode), respectively. All electrodes were attached with rubber bands.

In each session, a current of 2 mA was applied for 20 min (+10 s fade in and fade out periods). For sham stimulation, sinus (HW) mode was used for a duration of 30 s. The stimulation parameters are in accordance with established tDCS safety guidelines (e.g., Bikson et al., 2017).

### Equipment and stimuli

Participants were seated in a dimly lit room in front of a computer screen with a refresh rate of 60 Hz at a standardized distance of approximately 55 cm. During the experiment button presses were performed with the right index finger using a custom-made electromagnetic passive button. In active conditions, participants pressed the button actively themselves. In passive conditions, the button was pulled down automatically by an electromagnet with a maximum force of 4N. Participants’ fingers were tied to the button with an elastic fabric band to ensure that it would smoothly follow the movement of the button in passive conditions.

When a button press was registered by the computer (i.e., the button reached the lowest position) the presentation of a visual or an auditory stimulus was triggered. The visual stimulus was composed of a Gabor patch (1° visual angle, spatial frequency: 2 cycles/degree) presented at the center of the screen. The auditory stimulus was a brief sine-wave tone (2000 Hz with 2 ms rise and fall) played through headphones. Both stimuli were presented for a duration of 33.4 ms each. Stimuli were created and presented using Octave and the Psychophysics Toolbox (Brainard, 1997).

To ensure that sensory outcome perception would not be influenced by direct visual or auditory feedback of the actual button press movements, the button was covered by a black box and pink noise was applied through the headphones at individually adjusted volume during the whole experiment.

### Experimental design and task description

In each session, participants underwent multiple pairs of adaptation and test phases. Adaptation phases consisted of 18 consecutive button presses each followed by the presentation of the visual sensory outcome. Throughout an adaptation phase all button presses had to be performed either actively or were elicited passively (factor *movement type*). Importantly, the visual outcome occurred either directly after the button press was registered (0 ms delay) or after a constant delay of 150 ms (factor *adaptation delay*). While the 0 ms condition was assumed to match the natural expectation of temporal congruence between action and visual action-outcome (in active conditions) or between tactile or proprioceptive and visual sensory signals (in passive conditions), the constant delay was assumed to induce a prediction error and thus to trigger sensorimotor or inter-sensory temporal recalibration, respectively.

Each adaptation phase was followed by a test phase assessing the impact of the preceding adaptation phase on sensory perception. A test phase was composed of six individual test trials. In each trial the button was pressed once, either actively or passively (the movement type in each of the six test trials was identical to the one in the preceding adaptation phase). The button press triggered the presentation of either the visual or the auditory stimulus in each of the six test trials (factor *test modality*). In each trial one of six temporal delays (0, 83, 167, 250, 333, 417 ms; presented in frames: 0, 5, 10, 15, 20, 25) was inserted between button press and stimulus presentation. Thus, each delay appeared once in each test phase. Participants’ task was to report whether they detected a delay between the button press and its sensory outcome by pressing one of two keys on the keyboard with their left hand. The order of delays was counterbalanced across test phases and the assignment of keys was counterbalanced across participants. The TRE was defined as the difference in the percentage of detected delays in this task following an adaptation phase with vs. without constant outcome delay. Worse delay detection performance after adaptation with the delay of 150 ms would reflect a shift of the expected timing of the sensory stimulus in the direction of the delay and thus temporal recalibration.

In summary, the factors *adaptation delay* (0 vs. 150 ms), *movement type* (active vs. passive) and *test modality* (visual vs. auditory) were combined to eight different experimental conditions. Together with the factor *stimulation* (anodal vs. cathodal vs. dual-hemisphere vs. sham ctDCS) this resulted in a 4 × 2 × 2 × 2 within-subjects design.

### Procedure

Each participant went through all four stimulation conditions in four separate sessions. Intraindividual sessions were performed at least 24 h apart to prevent spill-over effects from the previous session. The order of stimulation conditions was counterbalanced across participants.

**FIGURE 1:**
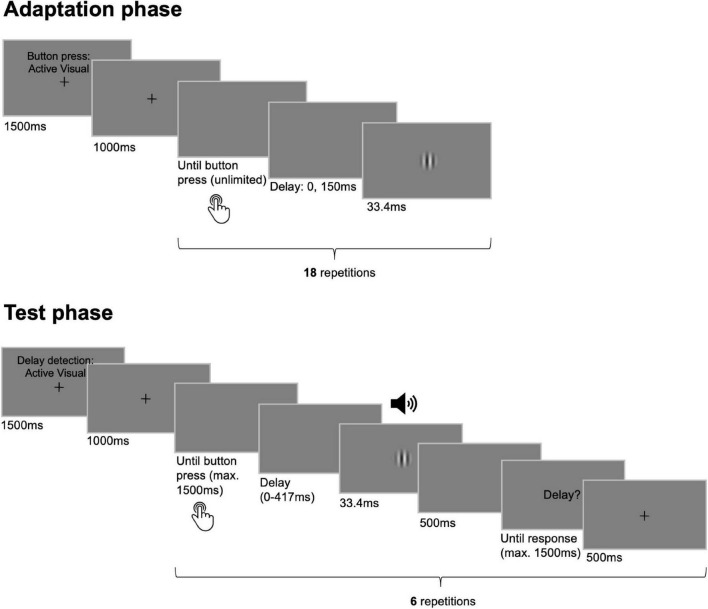
Trial sequence and timing of events. Participants went through multiple pairs of adaptation and test phases. During adaptation phases 18 button presses had to be performed either actively or they were elicited passively. Each button press was followed by the presentation of the visual outcome that appeared either undelayed with respect to the button press or that was delayed by 150 ms. In the following test phases composed of six test trials each, participants pressed the button once actively, or the button press was initiated passively. Here, the outcome followed the button press with one of six delay levels (0–417 ms) and participants’ task was to judge the presence of a delay in each trial. While the sensory modality of the outcome was always visual during adaptation phases it could be visual or auditory during test phases.

Each adaptation phase started with written instructions on the screen about the movement type of the upcoming button presses displayed for 1500 ms (see Figure 1). The instructions were displayed together with a fixation cross in the center of the screen which disappeared after another second indicating that participants could start pressing the button or that the button started to move passively. During the adaptation phase, each button press triggered the presentation of the visual outcome that was either undelayed with respect to the button press or that was delayed by 150 ms. Actively generated button presses in the adaptation phases were trained to be performed in an interval of approximately 750 ms and with a duration of 500 ms. The button press duration in both adaptation and test phases was chosen to be larger than the maximum delay inserted between button press and outcome (i.e., 417 ms) to prevent delay detection from being disturbed by the upwards movement of the button for any of the tested delay levels. To assure comparable button press parameters between active and passive movement conditions, passive button press intervals as well as durations dynamically adapted to the mean of the respective preceding active conditions throughout the experiment. Adaptation phases always terminated automatically after 18 button presses.

Each test phase also started with the fixation cross and instructions about the movement type and outcome modality of the upcoming test trials (displayed for 1500 ms). The disappearance of the fixation cross initiated the first of six test trials. In active conditions, participants had 1500 ms to press the button once. Yet, they were instructed to press the button not immediately after the fixation cross had disappeared but to withhold their button press action for about 700 ms. This was to ensure that the button press was not triggered as a reflex upon a starting signal but as a self-initiated action (Rohde and Ernst, 2013; van Kemenade et al., 2016; Straube et al., 2020). The same button press latency was applied for passive test trials. Each button press was followed by the presentation of the visual or auditory outcome with one of the six levels of temporal delay. After an interval of 500 ms the question “Delay?” was presented on the screen for a maximum of 1500 ms during which participants had to indicate via keypress whether they detected a delay between the button press and the sensory outcome. Afterward the fixation cross appeared again for 500 ms and its disappearance cued the beginning of the next test trial. The last trial of each test phase was followed by a jittered inter-trial interval before the beginning of the next adaptation-test pair (500, 1000, and 1500 ms).

In one session, each of the eight experimental conditions was presented in eight pairs of adaptation and test phases. Since each of the six delay levels was presented once in a test phase, this procedure provided us with eight trials per delay and condition for the analyses. Four adaptation-test pairs of each condition occurred in sequence in a first task block during which tDCS stimulation was applied. After the first task block, tDCS electrodes were detached during a short break. The remaining four adaptation-test pairs of each condition were presented in a second task block after the break in the same order as in the first task block. The second task block was completed without tDCS stimulation. Within each task block conditions with 0 and 150ms adaptation delay were blocked to prevent potential spill-over effects due to rapid switching. Whether the block of 0 or 150 ms delay was presented first as well as the order of conditions within these blocks was counterbalanced across participants.

After each session potential side effects of the tDCS stimulation (e.g., itching sensations, headache, changes in visual perception, difficulties in concentration) were assessed with a custom-designed questionnaire of 28 items using ratings on a scale from one (no side effect) to five (strong side effect). During the first session, participants additionally went through a training procedure before the beginning of the stimulation in order to familiarize with the task. They were instructed to place their finger loosely on the button and not to apply any counter-pressure in passive movement conditions. For the adaptation phases, they were trained to perform the button presses in intervals of approximately 750 ms and for a duration of 500 ms while receiving feedback about their performance. For the test phases, participants were trained in each of the experimental conditions, once with the undelayed and once with the maximally delayed sensory outcome (417 ms) to familiarize with the stimuli and the delays. Here, they were provided with feedback about the actual presence of a delay in the respective trial. Participants were asked to answer as accurately, not as fast as possible. Finally, they went through a 10-min training of the experiment to further familiarize with the task and the alternation of adaptation and test phases.

### Data analyses

Test trials for which no button press (0.8% of all trials) or response (1.4% of all trials) was registered were excluded from the analyses.

The proportion of detected test delays as a measure for delay detection performance was calculated separately for each participant and experimental condition. These data were then modeled by fitting psychometric functions in form of a cumulative Gaussian distribution function using the Psignifit toolbox version 4 (Schütt et al., 2016) for Python version 3.8.5 (Python Software Foundation^1^). Detection thresholds (i.e., the delay that was detected in 50% of all trials) and slopes (evaluated at the detection thresholds) were derived as summary measures from the psychometric functions. While the detection thresholds serve as a measure for the general delay detection performance (with lower values indicating better performance), the slopes represent the increment in detected delays when the amount of delay increases and thus indicate the discrimination ability between delay levels.

In line with the pre-registered analysis plan^2^, repeated-measures ANOVAs were performed to examine effects of temporal recalibration. But to get a more direct insight on the influence of the experimental manipulations on recalibration, the adaptation delay was not included as a factor in the analysis, but the TRE was used as a dependent variable. The TRE was quantified as the difference in detection thresholds in conditions with the adaptation delay of 150 ms vs. 0 ms. Positive values indicate a rightward shift of the psychometric function which corresponds to a decrease in delay detection performance after adaptation to the 150 ms delay as expected for temporal recalibration. Further, to better differentiate between the influence of ctDCS on the TRE in both test modalities, two repeated-measures ANOVAs were performed separately for the visual and auditory test modality, each including the factors *stimulation* and *movement type*. Similarly, repeated-measures ANOVAs were performed on the difference in slopes of conditions with 0 ms vs. 150 ms adaptation delay since temporal recalibration might also result in a lower ability to discriminate between delay levels represented in flatter slopes of the psychometric functions. *Post hoc* two-samples *t*-tests were used for significant main and interaction effects to inspect differences between movement types and stimulation conditions compared to the sham control condition. Additionally, in case of significant differences in the TRE between conditions, one-sample *t*-tests were further used to examine whether the respective TREs differed significantly from zero. As the TRE is defined as decreased delay detection performance after exposure to the adaptation delay of 150 ms, one-tailed *t*-tests were used. For effects pointing in the negative direction, only descriptive results are reported. All ANOVAs and *t*-tests were conducted with JASP (Version 0.14.1; JASP Team, 2020).

Although the TRE has often been defined in terms of a shift in detection thresholds of psychometric functions fitted to the delay detection rates (Stetson et al., 2006; Stekelenburg et al., 2011; Tsujita and Ichikawa, 2012; Rohde and Ernst, 2013; Cai et al., 2018; Arikan et al., 2021), this analysis is limited to the fact that only the shift in the overall detection performance across all assessed delay levels is considered. However, it is also conceivable that shifts in detection performance after exposure to the constant adaptation delay are constrained to a specific delay range and might occur, e.g., only at the most uncertain delays or at delays which are close to the adaptation delay. After temporal recalibration these specific delays might be less likely to be detected even without a general shift in detection thresholds.

In that sense, we further explored the distribution of the TRE across the tested delay levels by generalized estimating equations (GEE) analyses using IBM SPSS Statistics (Version 25.0). Here, the difference in the percentage of detected delays between 150 vs. 0 ms adaptation delay conditions served as measure for the TRE which can be computed separately for each test delay level. For the regression coefficients an AR (1) working correlation structure and robust (sandwich) covariance estimators were used. The analysis was performed separately for visual and auditory test modalities and the respective models were composed of the factors *stimulation* (anodal, cathodal, dual-hemisphere, sham ctDCS), *movement type* (active, passive), and *test delay* (0, 83, 167, 250, 333, 417 ms). A full factorial model was used with all main and interaction effects of the included factors and the TRE was modeled with a linear link function. If indicated, post-hoc tests for differences in TRE between active and passive conditions as well as between stimulation conditions and the sham control condition were then calculated to further explore the direction of effects. Since this latter analysis was only done exploratory to investigate the potential influence of the test delay in the emergence of the TRE and *post hoc* tests were only calculated for significant main and interaction effects, the *p*-values reported here are not corrected for multiple comparisons.

Finally, differences in perceived side effects between the ctDCS stimulation conditions were assessed by paired-samples *t*-tests using the average score of all questionnaire items.

## Results

### Effects of temporal recalibration

Repeated-measures ANOVAs were performed on the TRE (difference in detection thresholds of 150 ms vs. 0 ms adaptation delay conditions) for each test modality (see section 2 in Supplementary material for a full overview of all effects of these analyses). For the visual modality, no main or interaction effects reached significance (all *p* > 0.43) suggesting that ctDCS did not influence the visuomotor or visuo-tactile TRE. Notably, according to one-sample *t*-tests, the TREs in the visual modality were also not significantly greater than zero in the sham control condition, neither the visuomotor TRE in active [*M* = 11.518, *SD* = 41.310, *t*(21) = 1.308, *p* = 0.103, *d* = 0.279, one-tailed], nor the visuo-tactile TRE in passive conditions [*M* = –12.439, *SD* = 58.020].

**FIGURE 2:**
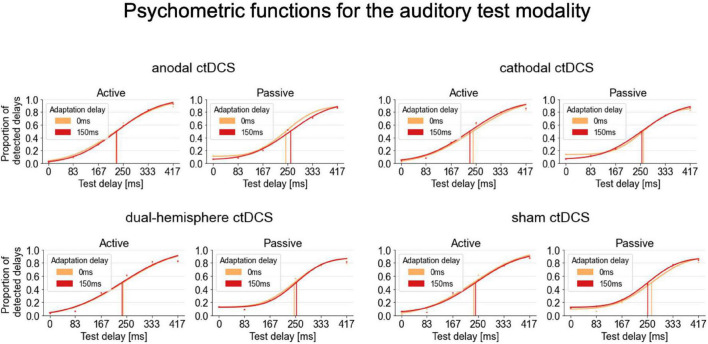
Psychometric functions fitted to the delay detection data for the auditory test modality. Group psychometric functions are displayed separately for each stimulation condition, movement type and adaptation delay. A TRE corresponds to a rightward shift of the psychometric functions for the 150 ms (red) compared to the 0 ms (orange) adaptation delay condition indicating decreased delay detection performance and thus temporal recalibration. Psychometric functions are displayed on group level only for illustration purposes. For the statistical analyses, the functions were fitted to and summary measures were derived from participants’ individual detection rates.

**FIGURE 3:**
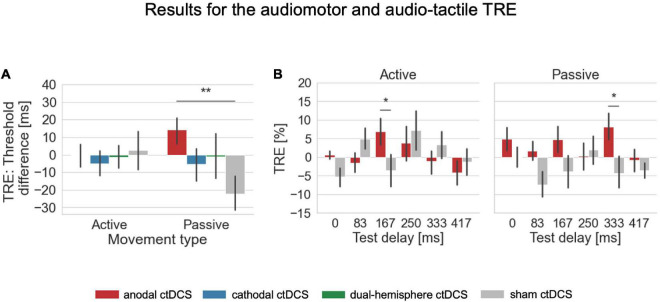
Results for the audiomotor and audio-tactile TRE. **(A)** For the overall shift in detection performance after temporal recalibration, the TRE was defined as the difference in detection thresholds derived from the fitted psychometric functions in conditions with the adaptation delay of 150 ms vs. 0 ms (where positive values indicate a rightward shift of the psychometric functions and thus worse performance after exposure to the 150ms delay indicating temporal recalibration into the expected direction). The audio-tactile TRE (in passive conditions) was here facilitated by anodal ctDCS compared to the sham control condition. **(B)** For a further exploration of the TRE across individual tested delay levels, the TRE was defined separately for each test delay as the difference in the percentage of detected delays between 0 and 150 ms adaptation delay conditions. For the audiomotor TRE (in active conditions), a facilitation by anodal ctDCS compared to sham occurred only for the test delay of 167 ms which was the one closest to the adaptation delay. For the audio-tactile TRE, the faciliatory effect was strongest at the larger test delay level of 333 ms. There were no significant main or interaction effects in both types of analyses on the TRE in the visual modality. Error bars indicate standard errors of the means. *p < 0.05, **p < 0.01.

For the auditory test modality, the interaction of *stimulation* and *movement type* was significant [*F*(3,63) = 2.810, *p* = 0.047, η_*p*_^2^ = 0.118; see Figure 2 for an overview of the psychometric functions fitted for these conditions]. According to post-hoc paired-samples *t*-tests, in passive conditions, anodal ctDCS increased the audio-tactile TRE compared to sham ctDCS [*t*(21) = 3.090, *p* = 0.006, *d* = 0.659, two-tailed; according to a Bonferroni corrected alpha of 0.05/3 (i.e., corrected for the three comparisons of ctDCS vs. sham) = 0.016; see Figure 3A]. More precisely, as further explored by one-sample t-tests, the audio-tactile TRE after anodal ctDCS was significantly greater than zero [*M* = 14.174, *SD* = 36.473, *t*(21) = 1.823, *p* = 0.041, *d* = 0.389, one-tailed], but was absent after sham ctDCS [*M* = –22.293, *SD* = 46.460].

No other stimulation condition elicited a TRE significantly different from the sham control condition for neither passive [cathodal vs. sham ctDCS: *t*(21) = 1.522, *p* = 0.143, *d* = 0.324; dual-hemisphere vs. sham ctDCS: *t*(21) = 1.464, *p* = 0.158, *d* = 0.321] nor active movement conditions [anodal vs. sham ctDCS: *t*(21) = –0.232, *p* = 0.819, *d* = –0.049; cathodal vs. sham ctDCS: *t*(21) = –0.599, *p* = 0.556, *d* = –0.128; dual-hemisphere vs. sham ctDCS: *t*(21) = –0.390, *p* = 0.701, *d* = –0.083].

Repeated-measures ANOVAs on the difference in slopes of the psychometric functions (between 0 ms vs. 150 ms adaptation delay conditions) did not reveal any significant main or interaction effects for either of the two test modalities (visual: all *p* > 0.51, auditory: all *p* > 0.37). Note that results derived from an alternative analysis approach with similar GEE analyses as described in section “Data analyses” including the factors *stimulation* and *movement type* led to comparable findings for all reported effects.

### Distribution of temporal recalibration effects across test delays

We further explored stimulation-dependent modulations of the TRE across the delay levels used in the test phases by GEE analyses including the factors *test delay*, *stimulation*, and *movement type*. The TRE was here defined as difference in the percentage of detected delays between 150 and 0 ms adaptation delay conditions as this measure can be derived for each test delay level individually. Here, interaction effects including the factors *stimulation* and *test delay* were of interest (see section 3 in Supplementary material for a full overview of all effects of these analyses).

For the visual test modality, again, none of the interaction effects reached significance (all *p* > 0.11). The analyses for the auditory test modality revealed a significant interaction of *stimulation* and *test delay* [Wald Chi-Square (df = 15) = 82.104, *p* < 0.001] indicating that stimulation-dependent effects on the TRE differed here depending on the amount of temporal delay presented in the test phase. *Post hoc* tests specified that the facilitation of the TRE by anodal ctDCS compared to sham manifested for the test delay closest to the adaptation delay [167 ms, anodal vs. sham ctDCS: mean difference = 9.575, standard error = 3.527, df = 1, *p* = 0.007]. Again, the TRE was significantly greater than zero after anodal ctDCS for this delay level [*M* = 5.855, *SD* = 12.403, *t*(21) = 2.214, *p* = 0.019, *d* = 0.472, one-tailed] which was absent in the sham condition [*M* = –3.720, *SD* = 12.981]. Moreover, adaptation to the constant delay of 150 ms was associated with fewer false alarms (i.e., fewer delay responses for the undelayed condition) after anodal [0 ms, anodal vs. sham ctDCS: mean difference = 5.370, standard error = 2.497, df = 1, *p* = 0.031] and cathodal ctDCS [0 ms, cathodal vs. sham ctDCS: mean difference = 5.411, standard error = 2.752, df = 1, *p* = 0.049] compared to the sham control condition. TREs were significantly greater than zero after anodal as well as cathodal ctDCS at this delay level [anode: *M* = 2.719, *SD* = 6.213, *t*(21) = 2.053, *p* = 0.026, *d* = 0.438, one-tailed; cathode: *M* = 2.760, *SD* = 7.094, *t*(21) = 1.825, *p* = 0.041, *d* = 0.389, one-tailed], but not present after sham ctDCS [*M* = –2.652, *SD* = 9.776].

Finally, the interaction *stimulation × movement type × test delay* was significant [Wald Chi-Square (df = 15) = 37.183, *p* = 0.001]. Thus, we inspected the *post hoc* comparisons of anodal compared to sham ctDCS to investigate whether the faciliatory effects of anodal ctDCS differed across the range of test delays depending on the movement type. Accordingly, the facilitation of the effect for the 167 ms test delay by anodal ctDCS was specific to the audiomotor TRE in active movement conditions [active, 167 ms, anodal vs. sham ctDCS: mean difference = 10.498, standard error = 4.389, df = 1, *p* = 0.017; see Figure 3B]. According to a one-sample *t*-test, the audiomotor TRE after the anodal stimulation was significantly greater than zero [anode: *M* = 6.953, *SD* = 17.556, *t*(21) = 1.858, *p* = 0.039, *d* = 0.396, one-tailed; sham: *M* = –3.544, *SD* = 20.423], and did not differ significantly from the audio-tactile TRE induced for this delay level in passive conditions [anodal ctDCS, 167 ms, active vs. passive: mean difference = 2.197, standard error = 5.113, df = 1, *p* = 0.667].

In contrast, in passive movement conditions, the facilitation by anodal ctDCS occurred at a larger test delay level of 333 ms [passive, 333 ms, anodal vs. sham ctDCS: mean difference = 12.446, standard error = 5.081, df = 1, *p* = 0.014]. According to a one-sample *t*-test the audio-tactile TRE was significant here after anodal ctDCS [anode: *M* = 8.144, *SD* = 17.362, *t*(21) = 2.200, *p* = 0.020, *d* = 0.469, one-tailed; sham: *M* = –4.302, *SD* = 20.830] and differed also weakly from the audiomotor TRE for this delay level in active conditions [anodal ctDCS, 333 ms, active vs. passive: mean difference = –9.280, standard error = 5.160, df = 1, *p* = 0.072].

### Side effects of the stimulation

Overall, the magnitude of perceived side effects due to the tDCS stimulation was rated to be low [anodal ctDCS: mean = 1.429, *SD* = 0.346; cathodal ctDCS: mean = 1.326; *SD* = 0.358; dual-hemisphere ctDCS: mean = 1.209, *SD* = 0.243; sham ctDCS: mean = 1.253; *SD* = 0.188]. The comparison of perceived side effects between the ctDCS conditions compared to the sham control condition revealed a significant difference for anodal ctDCS [*t*(21) = 3.106, *p* = 0.005, *d* = 0.662, two-tailed; according to a Bonferroni corrected alpha of 0.05/6 (i.e., corrected for the pair-wise comparisons of all stimulation conditions) = 0.008]. There were no significant differences for the comparison of sham stimulation against cathodal [*t*(21) = 1.089, *p* = 0.288, *d* = 0.232, two-tailed] or dual-hemisphere ctDCS [*t*(21) = –0.909, *p* = 0.373, *d* = –0.194, two-tailed]. Furthermore, ratings for perceived side effects were significantly higher after anodal compared to dual-hemisphere ctDCS [*t*(21) = 3.477, *p* = 0.002, *d* = 0.741, two-tailed]. However, control analysis including side effects as covariate of no interest suggest that main results cannot be explained by side effects alone (see section 4 in Supplementary material).

## Discussion

The complex and constantly changing sensory environment calls for flexible recalibration of the predicted timing between actions and their sensory outcomes. Since the neural underpinnings of sensorimotor temporal recalibration are largely unknown this study investigated the role of the cerebellum in this process by means of ctDCS. In an adaptation phase, participants were exposed to constant delays of 150 ms between actively or passively generated button presses and visual sensory outcomes while receiving either anodal, cathodal, dual-hemisphere or sham ctDCS. A delay detection task assessed if and to what extent the constant delays triggered a TRE in the visual and auditory modality. The results indicated that the exposure to constant delays did not result in a visuomotor or visuo-tactile TRE. However, a TRE occurred after anodal ctDCS in the auditory modality that was absent in the sham control condition. This effect was differentially distributed across the delay levels in the delay detection task depending on the movement type. In active conditions, anodal ctDCS facilitated the audiomotor TRE compared to sham stimulation at the delay level closest to the delay during the adaptation phase (167 ms), but at a larger delay level (333 ms) for the audio-tactile TRE in passive conditions.

These findings suggest a general role of the cerebellum in potentially supra-modal temporal recalibration across sensorimotor and perceptual domains, but the differential manifestation of the effect across tested delay levels in active and passive conditions point to differences in the underlying mechanisms depending on the availability of action-based predictions.

### Effects of cerebellar transcranial direct current stimulation on temporal recalibration depending on the movement type (active versus passive)

We expected ctDCS to influence action-based prediction mechanisms in the cerebellum and therefore the TRE in both visual and auditory modalities specifically in active movement conditions. Indeed, in the auditory test modality anodal ctDCS facilitated the occurrence of the TRE compared to the sham control condition. Surprisingly however, this stimulation-dependent effect was not specific to active conditions, but an audio-tactile TRE appeared across the tested delay levels only in passive conditions. However, an inspection of the faciliatory effect of anodal ctDCS for the individual test delay levels revealed that in active conditions an audiomotor TRE could be elicited by anodal ctDCS specifically at the 167 ms delay which was the delay level closest to the constant delay of 150 ms used during the adaptation phases. In contrast, in passive conditions, a significant facilitatory effect of anodal ctDCS on the audio-tactile TRE occurred at the later 333 ms delay level, at which the influence of the same stimulation on the audiomotor TRE had already decreased.

This raises the question of why the effect of ctDCS generally extended to inter-sensory temporal recalibration and therefore also appeared in passive conditions, and what reason might underlie the different manifestation of the effects across the range of tested delays depending on the movement type.

#### Facilitation of temporal recalibration by anodal cerebellar transcranial direct current stimulation is not limited to the sensorimotor context

Since anodal tDCS is known to enhance cortical excitability (Nitsche and Paulus, 2000) the stronger recalibration effects observed in this stimulation condition are likely to be attributed to the facilitation of cerebellar-dependent mechanisms underlying temporal recalibration, e.g., by increasing the sensitivity to temporal prediction errors. Even though this faciliatory effect was distributed differently across the tested delay levels in active and passive conditions, the fact that it was generally not limited to the active conditions, as we originally expected, but occurred even stronger in passive ones, suggests a role of the cerebellum not exclusively in sensorimotor but also in inter-sensory temporal recalibration.

Indeed, multiple studies support the involvement of the cerebellum in a variety of processes beyond sensorimotor functions ranging from perceptual processing (Baumann et al., 2015) and performance monitoring across domains of action, perception, and cognition (Peterburs and Desmond, 2016) to higher-level cognitive functions (Koziol et al., 2014). Traditionally, predictive processing in the cerebellum is often associated specifically with action-based predictions or with the detection of mismatches between expected and observed action-outcomes (Wolpert et al., 1998; Blakemore et al., 2001; Straube et al., 2017b; Arikan et al., 2019; van Kemenade et al., 2019). However, since cerebellar pathways do not only connect to motor areas, but also to a variety of other cortical areas including sensory regions (Strick et al., 2009; Sultan et al., 2012; Baumann et al., 2015), the cerebellum can be regarded as suitable for generating and updating predictive models in both motor and perceptual domains (Kotz et al., 2014). In this line it has been proposed that very similar mechanisms are at play for generating purely sensory predictions as for sensorimotor predictions (Schubotz, 2007) and that the posterior cerebellum completes similar tasks as the forward model in the action domain, but for perceptual processes with a timing aspect (O’Reilly et al., 2008). Indeed, the cerebellum has been shown to be relevant for temporal predictions also for action-independent sensory events and for the detection of mismatches between predicted and observed inter-sensory timing (Moberget et al., 2008; O’Reilly et al., 2008; Beudel et al., 2009; Coull et al., 2013; Kotz et al., 2014). In similar veins, although the cerebellum is commonly known for motor adaptation and thus for prediction adaptation in the action domain (Tseng et al., 2007; Synofzik et al., 2008; Izawa et al., 2012; Schlerf et al., 2012; Cao et al., 2017), recalibration of the expected timing of purely perceptual events could also be associated with the cerebellum, which suggests an important contribution to temporal recalibration of predictive models upon sensory prediction errors, but not exclusively for action-related processes (Roth et al., 2013).

Thus, in our study anodal ctDCS could have facilitated temporal recalibration particularly in an inter-sensory context by promoting the updating of the expected relative timing between tactile and auditory sensory signals. This adds evidence to the importance of the cerebellum for temporal recalibration of predictive mechanisms, however not exclusively within the motor domain as previously suggested (e.g., Cao et al., 2017), but also within the perceptual domain.

#### Anodal cerebellar transcranial direct current stimulation differentially impacts delay perception after temporal recalibration in the sensorimotor versus inter-sensory context

If anodal ctDCS facilitated the TRE identically in passive and active conditions, then this would suggest that the TRE in active conditions can be explained by the facilitation of inter-sensory recalibration mechanisms alone, since the additional availability of the motor commands would not provide any further explanatory contribution to the emergence of the effect in this condition. But notably, although anodal ctDCS facilitated the TRE in the auditory modality for both movement types, differences emerged in the manifestation of the effect when it is considered separately for the different delay levels used in the test phases. Thus, there may be differences in the exact underlying temporal recalibration mechanisms depending on the movement type.

For active conditions, the fact that the facilitation of the TRE by anodal ctDCS compared to sham occurred precisely at the delay level closest to the adaptation delay may point to a particularly accurate recalibration mechanism. This could be related to the fact that sensory action-outcomes are considered as particularly well predictable due to the availability of information on the motor commands (Blakemore et al., 1998; Wolpert et al., 1998; Elijah et al., 2016; Cao et al., 2017). It has been shown that this can result in sensory stimuli generated by self-induced actions being perceptually enhanced and leading to sharper neural representations compared to passively elicited sensory events (Yon et al., 2018). This is also reflected in our data in higher delay detection performance in active compared to passive conditions for auditory action-outcomes (see section 5 in Supplementary material). Such an enhanced perception of actively produced action-outcomes could also imply the generation of more precise error signals when a constant delay indicates a discrepancy between the temporal prediction and the actual occurrence of the outcome. Compared to passive conditions, the facilitation of cerebellar recalibration mechanisms could therefore also lead to a very precise shift in the prediction about the temporal occurrence of the action-outcome, affecting delay detection responses in the test phases only at the level close to the adapted delay. Thus, the facilitation of the audiomotor TRE by anodal ctDCS reaches significance only for the 167 ms delay while it decreases noticeably for larger delay levels.

Such a precise recalibration mechanism could be particularly important in a sensorimotor context, because in real-life situations, changes in the relative timing between actions and their outcomes have direct implications for motor behavior, as movements may need to be adjusted accordingly. Thus, precise sensorimotor temporal recalibration could be important to still enable accurate motor behavior under flexibly changing environmental conditions.

For passive conditions on the other hand, faciliatory effects of anodal ctDCS on the audio-tactile TRE appeared at larger delays (i.e., 333 ms). In part, this could be related to the fact that detection performance was generally worse in passive compared to active conditions. It might thus be that the perceptual performance at medium-size delay levels was generally too low for a strong recalibration effect to manifest here. Only at larger delay levels the recalibrated inter-sensory timing leads to significant decreases in delay detection performance. Nonetheless, the audio-tactile TRE induced by anodal ctDCS appears somewhat more distributed across the delay range, which becomes also apparent in the fact that the audio-tactile and audiomotor TRE do not differ significantly at the earlier 167 ms delay. But since the audiomotor TRE after anodal ctDCS decreases across delays after its early peak at the 167 ms delay, the relative impact of anodal ctDCS grows stronger for the audio-tactile TRE, resulting in significant differences between the audiomotor and audio-tactile TRE at the larger delay level of 333 ms. This somewhat broader distribution of the audio-tactile TRE after anodal ctDCS could also explain why it is overall, i.e., across all delay levels, stronger than the audiomotor TRE.

In conclusion, anodal ctDCS facilitated both the audio-tactile and the audiomotor TRE, but the differential manifestation of the effect across delay levels suggests the additional involvement of action-based predictive mechanisms for the emergence of the effect in active conditions potentially leading to a more precise audiomotor TRE. It should be noted though that these delay-dependent effects appeared to be rather weak and *post hoc* tests of this exploratory analysis are uncorrected for the multiple testing at each of the six delay levels and should therefore be interpreted with caution. Although the exact explanation for and interpretability of the differential manifestation of the sensorimotor and inter-sensory TRE as well as the overall rather weak recalibration effects in this study remains open, our findings nevertheless highlight the importance of not just focusing on the overall shift in perceptual thresholds (evaluated across all tested delay levels) during the investigation of temporal recalibration, but also at the exact manifestation of the effect at different delays.

### Effects of cerebellar transcranial direct current stimulation on temporal recalibration depending on the sensory modality (visual versus auditory)

Since predictions about sensory action-outcomes are assumed to be generated on a supra-modal level, we expected that a TRE would manifest after the visual recalibration procedure in the visual, but also in the auditory test modality in active movement conditions, and that anodal ctDCS would further boost these effects.

Surprisingly, however, without the influence of ctDCS, i.e., in the sham control condition, a TRE did not manifest for the visual modality in either active or passive conditions and did not transfer to the auditory modality either. Only after anodal ctDCS recalibration occurred in the auditory modality which was still absent in visual conditions. This raises the question of the reason for the resistance of the visual modality to behaviorally relevant recalibration in our study, as well as why the constantly delayed visual stimulus during adaptation phases nevertheless produced an auditory TRE after anodal ctDCS.

#### Resistance of the visual modality to temporal recalibration

Although recalibration of the perceived timing between actions and their visual sensory outcomes could be demonstrated in a range of previous studies (Cunningham et al., 2001; Heron et al., 2009; Keetels and Vroomen, 2012; Rohde and Ernst, 2013; Sugano et al., 2016; Aytemür et al., 2017; Arikan et al., 2021), a certain robustness of visual perception in the context of sensorimotor temporal recalibration has been reported earlier for instance by the absence of a behaviorally relevant visual sensorimotor TRE (Sugano et al., 2017). Additionally, an auditory sensorimotor TRE did often not transfer to the visual modality (Sugano et al., 2012; Arikan et al., 2021) while the opposite could frequently be demonstrated (Heron et al., 2009; Sugano et al., 2010, 2012; Arikan et al., 2021). Additionally, other phenomena based on similar sensorimotor prediction mechanisms like the intentional binding effect, i.e., an implicit measure for the experience of agency over action-outcomes, was found to be weaker for visual compared to auditory stimuli which might be related to the rather inaccurate time perception in the visual compared to the auditory modality (Ruess et al., 2018).

Similarly, in the context of inter-sensory timing, synchrony perception of visuo-tactile stimuli (as it is relevant for passive conditions in our study) has been shown to have rather low resolution as compared to, for example, audio-tactile stimuli (Fujisaki and Nishida, 2009). This implies that short temporal mismatches between visual and tactile sensory events might not be reliably detected and that the temporal relationship between both modalities could therefore be less prone to recalibration and its modulation by ctDCS (Hanson et al., 2008; but see: Harrar and Harris, 2008; Keetels and Vroomen, 2008).

Thus, in our study, the short temporal lag of 150 ms during the adaptation phase could have caused too noisy or unreliable prediction errors in the visual modality to induce recalibration of upcoming sensorimotor predictions or recalibration of inter-sensory timing and thus a behaviorally observable TRE in active or passive movement conditions. Nevertheless, we cannot rule out the possibility that the effects of temporal recalibration in general, as well as the effect of ctDCS on temporal recalibration, were so small for the visual modality that we are lacking sufficient statistical power in this study to detect these effects.

#### Facilitation of the temporal recalibration effect in auditory conditions suggests a supra-modal mechanism

The emergence of an audiomotor and audio-tactile TRE even though the sensory modality during the adaptation phase was visual would point to the recalibration of predictive or perceptual mechanisms that operate on a supra-modal level and not in modality-specific circuits. The fact that a TRE occurred indeed only in the auditory modality after anodal ctDCS in our study might be related to a generally more precise temporal perception and discrimination performance for auditory stimuli (Grondin, 2010; Grahn, 2012). This also implies a higher predictability of the temporal occurrence of auditory stimuli and predictability of sensory signals is known to be important for temporal recalibration to occur (Rohde et al., 2014).

Thus, our results suggest that the delayed visual stimulus in the adaptation phases still resulted in an updating of the perceived relative timing between the active or passive button movement and the feedback presentation, even though its effects were not measurable as TRE in the visual modality possibly because of the rather imprecise temporal perceptual acuity. However, with the faciliatory influence of anodal ctDCS, prediction error processing increased and resulted in a TRE now assessable in the auditory modality possibly due to the temporally more accurate perception.

Notably, we originally expected that anodal ctDCS would mainly promote the modality-transfer of the TRE in active conditions due to the facilitation of supra-modal sensorimotor recalibration mechanisms in the cerebellum, but the effect extended to passive conditions and thus to inter-sensory recalibration. Cross-modal transfer of the TRE in an inter-sensory context has also been shown in previous studies (di Luca et al., 2009; Arikan et al., 2021) and speaks here for general temporal recalibration mechanisms in the cerebellum that operate on a supra-modal level.

Nevertheless, it should be noted that due to the absence of effects in the visual modality conclusions about supra-modal recalibration mechanisms should be made with caution. Still, the fact that the visuomotor and visuo-tactile recalibration procedure could trigger an audiomotor or audio-tactile TRE, respectively, points to recalibration mechanisms in the cerebellum evoked by anodal ctDCS that are not modality specific.

### Limitations and outlook

Some limitations of the study design and interpretability of the results should be considered. The potential of ctDCS in affecting performance in a variety of tasks has been demonstrated in an increasing number of studies (Grimaldi et al., 2016). Also in our study, anodal ctDCS appeared to facilitate recalibration effects in the auditory modality. It has to be noted though that the spatial resolution of this stimulation technique is known to be rather low. Therefore, we do not know exactly which parts of the cerebellum were targeted by the stimulation in our study, whether the effect was similar across subjects, or whether the stimulation effects have extended to nearby areas. At the same time, the anatomy and functionality of the cerebellum are very complex and different cerebellar areas are involved in different motor, perceptual or cognitive processes (Koziol et al., 2014; Baumann et al., 2015). This issue may explain why there were rather large individual differences in the obtained effects (as it becomes evident by the large standard errors) and may also provide an explanation for why we found effects of anodal ctDCS not only in active but also in passive conditions, since parts of the cerebellum, i.e., the posterior cerebellum, have been suggested as particularly important for recalibration in a purely sensory context (Roth et al., 2013). The use of high-definition tDCS may help to achieve more spatially defined effects in the future and thus to further disentangle contributions of the cerebellum to sensorimotor and inter-sensory temporal recalibration, respectively.

Furthermore, although anodal tDCS is generally thought to increase cortical excitability, the evidence for polarity-dependent effects of tDCS on the cerebellum is heterogeneous. While some studies find polarity-dependent effects in the expected direction (Galea et al., 2009; Jayaram et al., 2012), others failed to find a difference between anodal and cathodal ctDCS (Shah et al., 2013). This could be explained in part by the complex anatomy of the cerebellum, in which the orientation of neurons in different cerebellar areas may differ with respect to the induced electric fields (Grimaldi et al., 2016). Indeed, our findings don’t show a clear pattern with respect to polarity-specific effects, as only anodal ctDCS had an impact on temporal recalibration. Although the reason for this remains open, it cannot be excluded that the perceived side effects of the tDCS stimulation which were particularly prominent after anodal ctDCS contributed to the emergence of behavioral effects specifically in this condition. Nonetheless, the specific and expected facilitation of recalibration effects by anodal ctDCS still argues for a polarity-dependent influence of the stimulation and therefore highlights anodal ctDCS as a potentially promising tool for modulating temporal recalibration in future research. However, the complex effects of this study also stress the importance of identifying additional brain areas next to the cerebellum which are involved in temporal recalibration mechanisms, e.g., by using fMRI, and that could constitute promising stimulation sites in future research on this topic.

It has to be noted that the recalibration effects in our study were overall rather small and not present without the facilitating influence of anodal ctDCS, i.e., in the sham control condition. The audio-tactile TRE in passive conditions seems to point even into the opposite direction than one would expect. Even though the reason for this remains unclear based on our data, considerably smaller shifts in temporal perception during recalibration compared to the magnitude of the actual adaptation delay has been reported previously (e.g., Stetson et al., 2006). The particularly weak effects in this study may indicate features in our experimental design that made pronounced temporal recalibration difficult to occur.

First, the size of the recalibration effect, or even the occurrence of the effect itself, could be related to the amount of attention deployed to the sensory stimuli during adaptation. For example, it has been reported that awareness of the constant delay between action and visual outcome is necessary to trigger the visual sensorimotor TRE (Tsujita and Ichikawa, 2016). Furthermore, the magnitude of recalibration to an asynchrony between visual and auditory sensory signals has been shown to increase particularly when attention is explicitly directed to the temporal relationship between the sensory stimuli as opposed to other stimulus features (Heron et al., 2010). In our study, we asked participants to pay close attention to the sensory stimuli also during the adaptation phases. However, we cannot directly infer how much attention the sensory stimuli actually received, as participants had no other task during adaptation than to press the button actively or let it be passively moved and to observe the visual stimuli. Thus, it is unclear whether too little attention to the stimuli during adaptation can contribute to an explanation of the small recalibration effects. In future studies, attention to the sensory stimuli could be more explicitly controlled by, for example, implementing an oddball task during the adaptation phase that requires close attention to the stimuli to complete the task.

Second, it could be argued that recalibration effects that have built up over the course of an adaptation phase decay relatively quickly over the course of a test phase, so that the last trials of the test phase are less affected by recalibration, resulting in lower overall recalibration effects. To counteract this, previous studies have used so-called top-up trials, in which subjects were quickly exposed to the adaptation delay again before each test trial (e.g., Rohde and Ernst, 2013). However, since our test phase consisted of only six test trials followed by another adaptation phase, we think it is unlikely that effects of recalibration wore off so quickly and a similar experimental procedure has also reliably led to recalibration effects before (Arikan et al., 2021).

Lastly, within our experimental procedure, conditions with the same adaptation delay (0 vs. 150 ms) were each presented in a block during and after stimulation, resulting in multiple changes of the adaptation delay within a session. While it was intended to challenge the flexibility of recalibration mechanisms with this procedure, it is conceivable that the more frequent switching between adaptation delays in our experiment caused spill-over effects, thereby decreasing the overall size of the TRE or the time window available for the effect to build up before the adaptation delay changed again might have been too short. However, the rapid recalibration effects that could be observed even on a trial-by-trial basis at least in the inter-sensory domain (van der Burg et al., 2015; Lange et al., 2018) argue against that. Thus, the fact that anodal ctDCS was still able to elicit behaviorally relevant recalibration underlines the role of the cerebellum in flexibly adapting to just such rapid changes in the temporal relationship between an action and its sensory outcome or between the senses.

The prospect that anodal ctDCS may improve sensorimotor adaptation, has implications for neurological and psychiatric disorders known to have impairments in this process and potentially also in related processes like motor adaptation and learning. Patients with cerebellar ataxia for instance show impairments in the ability to adapt to visuomotor perturbations (Tseng et al., 2007) and exhibit problems in locomotor control, i.e., gait ataxia (Ilg and Timmann, 2013). Since the potential of anodal ctDCS for improving certain motor functions in such patient groups has already been demonstrated (Benussi et al., 2015, 2021; Wang et al., 2021), it may also be a promising tool to support rehabilitation of sensorimotor adaptation mechanisms, which are of great importance for motor performance in a variety of everyday tasks. One critical example is locomotion, whose successful functioning depends to a great extent on temporal perceptual recalibration mechanisms (Gonzalez-Rubio et al., 2019) as well as cerebellar processes (Morton and Bastian, 2006; Jayaram et al., 2012; Jossinger et al., 2020). Our results highlight the importance of the cerebellum in recalibration of sensorimotor and perceptual timing and therefore also stress its promising role as target for non-invasive brain stimulation in the context of rehabilitation of locomotor adaptation functions in these patients. First studies already demonstrated the efficacy of anodal ctDCS on posture and gait in this patient group (Benussi et al., 2015), providing a promising basis for future investigations of the specific conditions under which ctDCS may lead to improvements and long-lasting effects also in processes such as locomotor adaptation.

Beyond that, patients with schizophrenia spectrum disorder have been shown to have dysfunctions in forward model based predictive mechanisms (Ford et al., 2001; Bartolomeo et al., 2020; Uhlmann et al., 2020). Such a dysfunction, and especially the failure to adequately recalibrate internal model predictions to changes in the environment may underly symptoms of auditory hallucinations, i.e., when one’s inner speech is misinterpreted as externally generated, or passivity phenomena, i.e., when outcomes of own actions are perceived as externally produced (Pynn and DeSouza, 2013). Therefore, based on the findings of this study, a promising avenue for future research is to investigate whether anodal ctDCS can improve recalibration mechanisms and thus symptomatology in patients with schizophrenia spectrum disorder.

## Conclusion

The present study investigated the impact of ctDCS on visuomotor and visuo-tactile temporal recalibration and the manifestation of its effect on visual and auditory delay detection performance. We demonstrated that anodal ctDCS facilitated the TRE in the auditory modality in both active and passive conditions which points to a general role of the cerebellum in temporal recalibration across sensorimotor and perceptual domains. The fact that the effect occurred in the auditory modality even though the adaptation modality was visual further suggests that this temporal recalibration mechanism operates on a supra-modal level. The differential manifestation of the effect across tested delay levels in active and passive conditions, however, indicates a more precise cerebellar-dependent temporal recalibration mechanism in a sensorimotor context possibly due to the additional recalibration of action-based predictions.

Together these findings emphasize the role of the cerebellum in potentially supra-modal temporal recalibration mechanisms in both perceptual and sensorimotor domains, but there might be differences in the precision of recalibration depending on the availability of action-based predictions. Additionally, despite the complex pattern of observed effects, our findings suggest that anodal ctDCS can be a promising tool for facilitating effects of temporal recalibration in the sensorimotor, but explicitly also in the inter-sensory domain.

## Data availability statement

The datasets presented in this study can be found in online repositories. The names of the repository/repositories and accession number(s) can be found below: doi: 10.5281/zenodo.6861087 (Zenodo), https://osf.io/qhryx (OSF preregistration).

## Ethics statement

The studies involving human participants were reviewed and approved by Ethics Commission of the Medical Faculty of University of Marburg, Germany. The patients/participants provided their written informed consent to participate in this study.

## Author contributions

BS and CS conceived and designed the study, and discussed and interpreted the data. CS was responsible for data acquisition and analysis, and wrote the manuscript. Both authors revised and approved the submitted version of the manuscript.

## Footnotes

1
https://www.python.org/

2
https://osf.io/qhryx
