# Supplementary material for: The impact of cerebellar transcranial direct current stimulation (tDCS) on sensorimotor and inter-sensory temporal recalibration
Source: Front Hum Neurosci. 2022 Aug 30;16:998843. doi: 10.3389/fnhum.2022.998843 (PMC9468227; doi:10.3389/fnhum.2022.998843)
Supplement: Supplementary file 1 [file Data_Sheet_1.docx]

**Supplementary material**

# **Power analysis**

The software MorePower 6.0.4 (Campbell & Thompson, 2012) was used to calculate the expected power for our main analysis, i.e., the 4 x 2 repeated-measures ANOVAs on the temporal recalibration effect (TRE) with the factors *stimulation* (anodal, cathodal, dual-hemisphere, sham ctDCS) and *movement type* (active, passive). We calculated the power for the 4 x 2 interaction effect which was of primary interest for our study. Previous studies on temporal recalibration (e.g., Arikan et al., 2021) or with a comparable task in a tDCS study design (Straube et al., 2017) reported large effects sizes with a comparable or even smaller sample size. Thus, considering our sample size of 22 participants and an expected effect size of η_p_^2^ = 0.2, this analysis results in an expected power of 91.2%. The effect size of η_p_^2^ = 0.2 corresponds to the average effect size of interaction effects found in a previous study of our lab with the same experimental design (Arikan et al., 2021). Using a more conservative effect size of η_p_^2^ = 0.14 which is by convention regarded as a large effect, still yields an expected power of 74.4%. Thus, based on effect sizes from previous studies, our sample size should provide us with sufficient power to find similar effects for the impact of cerebellar tDCS on our temporal recalibration experiment.

# **Supporting information for results on effects of temporal recalibration**

For investigating the TRE induced by the recalibration procedure, repeated-measures ANOVAs were performed for each test modality with the factors *stimulation* and *movement type* (see section 3.1 in manuscript). **Table 1** and **Table 2** provide an overview of the main and interaction effects of these analyses*.* The TRE as dependent variable was quantified as the difference delay detection thresholds for conditions with the adaptation delay of 150 vs. 0ms (with positive values indicating a rightward shift of the psychometric functions and thus decreased detection performance after exposure to the 150ms adaptation delay indicating temporal recalibration).

**Table 3** and **Table 4** summarize the results of the repeated-measures ANOVAs on the difference in slopes of the psychometric functions between 0ms vs. 150ms adaptation delay conditions.

**Table 1. Results of the ANOVA on the TRE (threshold differences) for the visual test modality**

| **Effect** | **df** | **F-value** | **p-value** | **η_p_^2^** |
| --- | --- | --- | --- | --- |
| stimulation | 3 | .565 | .640 | .026 |
| movement type | 1 | .628 | .437 | .029 |
| stimulation * movement type | 3 | .840 | .477 | .038 |

**Table 2. Results of the ANOVA on the TRE (threshold differences) for the auditory test modality**

| **Effect** | **df** | **F-value** | **p-value** | **η_p_^2^** |
| --- | --- | --- | --- | --- |
| stimulation | 3 | 1.416 | .246 | .063 |
| movement type | 1 | .074 | .788 | .004 |
| **stimulation * movement type** | **3** | **2.810** | **.047** | **.118** |

**Table 3. Results of the ANOVA on slope differences for the visual test modality**

| **Effect** | **df** | **F-value** | **p-value** | **η_p_^2^** |
| --- | --- | --- | --- | --- |
| stimulation | 3 | .399 | .754 | .019 |
| movement type | 1 | .446 | .511 | .021 |
| stimulation * movement type | 3 | .778 | .511 | .036 |

**Table 4. Results of the ANOVA on slope differences for the auditory test modality**

| **Effect** | **df** | **F-value** | **p-value** | **η_p_^2^** |
| --- | --- | --- | --- | --- |
| stimulation | 3 | 1.064 | .371 | .048 |
| movement type | 1 | .007 | .933 | < .001 |
| stimulation * movement type | 3 | .798 | .499 | .037 |

# **Supporting information for results on the distribution of temporal recalibration effects across test delays**

To explore the distribution of the TRE across the tested delay levels, GEE analyses were calculated for each test modality with the TRE as dependent variable including the factors *stimulation*, *movement type* and *test delay* (see section 3.2 in manuscript). **Table 5** and **Table 6** provide an overview of all effects of these analyses. Again, post-hoc tests were calculated for significant main and interaction effects of interest to quantify differences between active and passive conditions and between stimulation conditions and the sham control condition (see **Table 7**).

**Table 5. Results of the GEE analysis on the TRE across test delays for the visual test modality**

| **Effect** | **Wald-Chi-Square** | **df** | **p-value** |
| --- | --- | --- | --- |
| stimulation | 4.422 | 3 | .219 |
| movement type | .022 | 1 | .881 |
| **test delay** | **24.263** | **5** | **< .001** |
| stimulation * movement_type | 4.638 | 3 | .200 |
| stimulation * test delay | 21.595 | 15 | .119 |
| movement type * test delay | 4.306 | 5 | .506 |
| stimulation * movement type * test delay | 21.366 | 15 | .125 |

**Table 6. Results of the GEE analysis on the TRE across test delays for the auditory test modality**

| **Effect** | **Wald-Chi-Square** | **df** | **p-value** |
| --- | --- | --- | --- |
| stimulation | 7.528 | 3 | .057 |
| movement type | .031 | 1 | .860 |
| test delay | 6.133 | 5 | .293 |
| stimulation * movement_type | 5.777 | 3 | .123 |
| **stimulation * test delay** | **82.104** | **15** | **< .001** |
| movement type * test delay | 10.458 | 5 | .063 |
| **stimulation * movement type * test delay** | **37.183** | **15** | **.001** |

**Table 7. Post-hoc tests for significant interaction effects for the GEE analysis on the auditory test modality**

|  | **Mean diff.** | **Std. error** | **df** | **p-value** | *95% Wald confidence interval for diff.* | |
| --- | --- | --- | --- | --- | --- | --- |
|  |  |  |  |  | **low** | **high** |
| **stimulation * test delay** | | | | | | |
| **0ms: anodal vs. sham ctDCS** | **5.371** | **2.497** | **1** | **.031** | **.477** | **10.265** |
| **0ms: cathodal vs. sham ctDCS** | **5.411** | **2.751** | **1** | **.049** | **.018** | **10.804** |
| 0ms: dual-hem. vs. sham ctDCS | 2.164 | 2.775 | 1 | .435 | -3.275 | 7.604 |
| 83ms: anodal vs. sham ctDCS | 1.326 | 2.933 | 1 | .651 | -4.423 | 7.075 |
| 83ms: cathodal vs. sham ctDCS | 1.542 | 2.962 | 1 | .602 | -4.263 | 7.347 |
| 83ms: dual-hem. vs. sham ctDCS | 1.339 | 3.248 | 1 | .680 | -5.027 | 7.705 |
| **167ms: anodal vs. sham ctDCS** | **9.575** | **3.527** | **1** | **.007** | **2.661** | **16.489** |
| 167ms: cathodal vs. sham ctDCS | 3.192 | 3.508 | 1 | .363 | -3.683 | 10.068 |
| 167ms: dual-hem. vs. sham ctDCS | 3.068 | 4.535 | 1 | .499 | -5.821 | 11.957 |
| 250ms: anodal vs. sham ctDCS | -2.513 | 4.194 | 1 | .549 | -10.734 | 5.707 |
| 250ms: cathodal vs. sham ctDCS | -6.277 | 4.628 | 1 | .175 | -15.348 | 2.794 |
| 250ms: dual-hem. vs. sham ctDCS | -1.447 | 3.592 | 1 | .687 | -8.487 | 5.592 |
| 333ms: anodal vs. sham ctDCS | 3.964 | 3.040 | 1 | .192 | -1.994 | 9.922 |
| 333ms: cathodal vs. sham ctDCS | -.582 | 4.598 | 1 | .899 | -9.595 | 8.431 |
| 333ms: dual-hem. vs. sham ctDCS | -2.408 | 3.257 | 1 | .460 | -8.791 | 3.976 |
| 417ms: anodal vs. sham ctDCS | -.081 | 2.561 | 1 | .975 | -5.100 | 4.938 |
| 417ms: cathodal vs. sham ctDCS | -.798 | 2.809 | 1 | .776 | -6.304 | 4.708 |
| 417ms: dual-hem. vs. sham ctDCS | 2.083 | 3.522 | 1 | .554 | -4.820 | 8.987 |
| **stimulation * movement type * test delay** | | | | | | |
| 0ms, active: anodal vs. sham ctDCS | 5.763 | 3.093 | 1 | .062 | -.299 | 11.825 |
| **167ms, active: anodal vs. sham ctDCS** | **10.498** | **4.389** | **1** | **.017** | **1.896** | **19.099** |
| 83ms, passive: anodal vs. sham ctDCS | 9.064 | 4.895 | 1 | .064 | -.530 | 18.657 |
| 167ms, passive: anodal vs. sham ctDCS | 8.652 | 4.838 | 1 | .074 | -.829 | 18.134 |
| **333ms, passive: anodal vs. sham ctDCS** | **12.446** | **5.081** | **1** | **.014** | **2.487** | **22.404** |
| **83ms, passive: cathodal vs. sham ctDCS** | **8.441** | **4.003** | **1** | **.035** | **.595** | **16.288** |
| **0ms, active: dual-hem. vs. sham ctDCS** | **5.357** | **2.499** | **1** | **.032** | **.459** | **10.255** |
| 250ms, active: dual-hem. vs. sham ctDCS | -8.333 | 4.971 | 1 | .094 | -18.076 | 1.410 |
| **333ms, active: dual-hem. vs. sham ctDCS** | **-6.602** | **2.377** | **1** | **.005** | **-11.260** | **-1.943** |
| 0ms, sham ctDCS: active vs. passive | -5.086 | 3.051 | 1 | .095 | -11.067 | .894 |
| **83ms, sham ctDCS: active vs. passive** | **12.338** | **4.777** | **1** | **.010** | **2.975** | **21.701** |
| 333ms, anodal ctDCS: active vs. passive | -9.280 | 5.160 | 1 | .072 | -19.394 | .834 |
| **0ms, cathodal ctDCS: active vs. passive** | **-11.364** | **4.706** | **1** | **.016** | **-20.587** | **2.140** |

*Note.* For the three-way interaction, only tests at p < .10 are displayed for clarity.

# **Control analysis including stimulation side effects as covariate**

Since significant differences in perceived side effects were found for anodal ctDCS compared to the sham control condition and compared to dual-hemisphere ctDCS (see section 3.3 in manuscript), we included stimulation side effects as covariate into the GEE analysis on the TRE for the auditory test modality with the factors *stimulation*, *movement type*, *test delay* (which is responsible for the main results of this study). Accordingly, the main results of this analysis cannot be attributed to side effects alone, as the interaction effects of interest (i.e., the two-way interaction of *stimulation* and *test delay* as well as the three-way interaction of *stimulation*, *movement type* and *test delay*) still reach significance (see **Table 8**).

**Table 8. Results of the GEE analysis on the TRE across test delays for the auditory test modality including stimulation side effects as covariate**

| **Effect** | **Wald-Chi-Square** | **df** | **p-value** |
| --- | --- | --- | --- |
| stimulation | 2.260 | 3 | .520 |
| movement type | .346 | 1 | .556 |
| test delay | 3.522 | 5 | .620 |
| side effects | .316 | 1 | .574 |
| stimulation * movement_type | 1.273 | 3 | .736 |
| **stimulation * test delay** | **33.829** | **15** | **.004** |
| stimulation * side effects | 2.420 | 3 | .490 |
| movement type * test delay | 7.532 | 5 | .184 |
| movement type * side effects | .342 | 1 | .559 |
| test delay * side effects | 1.641 | 5 | .896 |
| **stimulation * movement type * test delay** | **45.065** | **15** | **< .001** |
| stimulation * movement type * side effects | .860 | 3 | .835 |
| **stimulation * test delay * side effects** | **62.307** | **15** | **< .001** |
| movement type * test delay * side effects | 9.459 | 5 | .092 |
| **stimulation * movement type * test delay * side effects** | **54.293** | **15** | **< .001** |

# **Differences in delay detection performance between active and passive movement types**

To explore differences in delay detection performance between active and passive movement types, we compared the detection thresholds derived from the psychometric functions between active and passive conditions and separately for each test modality by means of paired-samples t-tests. Results for the auditory test modality revealed that smaller delays could be detected for active [*M* = 239.192, *SD* = 93.183] compared to passive conditions [*M* = 259.744, *SD* = 94.633; *t*(21) = -2.470, *p* = .022, *d* = -.527, two-sided; ] indicating that self-generated action-outcomes were perceptually enhanced. For the visual test modality, there was no difference in delay detection thresholds between active [*M* = 243.810, *SD* = 89.473] and passive movement types [*M* = 243.765, *SD* = 94.444; *t*(21) = .005, *p* = .996, *d* = .001].

**References**

Arikan, B. E., van Kemenade, B. M., Fiehler, K., Kircher, T., Drewing, K., & Straube, B. (2021). Different contributions of efferent and reafferent feedback to sensorimotor temporal recalibration. *Scientific Reports*, *11*(1), 1–15. https://doi.org/10.1038/s41598-021-02016-5

Campbell, J. I. D., & Thompson, V. A. (2012). MorePower 6.0 for ANOVA with relational confidence intervals and Bayesian analysis. *Behavior Research Methods*, *44*(4), 1255–1265. https://doi.org/10.3758/s13428-012-0186-0

Straube, B., Schülke, R., Drewing, K., Kircher, T., & van Kemenade, B. M. (2017). Hemispheric differences in the processing of visual consequences of active vs. passive movements: a transcranial direct current stimulation study. *Experimental Brain Research*, *235*(10), 3207–3216. https://doi.org/10.1007/s00221-017-5053-x
